# The Third Transmembrane Domain of EscR Is Critical for Function of the Enteropathogenic *Escherichia coli* Type III Secretion System

**DOI:** 10.1128/mSphere.00162-18

**Published:** 2018-07-25

**Authors:** Irit Tseytin, Adi Madar, Bosko Mitrovic, Wanyin Deng, B. Brett Finlay, Neta Sal-Man

**Affiliations:** a Shraga Segal Department of Microbiology, Immunology and Genetics, Faculty of Health Sciences, Ben-Gurion University of the Negev, Be’er Sheva, Israel; b Michael Smith Laboratories, University of British Columbia, Vancouver, British Columbia, Canada; University of Rochester

**Keywords:** enteropathogenic E. coli, export apparatus, inner membrane proteins, transmembrane domain, type III secretion system

## Abstract

Many Gram-negative bacterial pathogens utilize a specialized protein delivery system, called the type III secretion system (T3SS), to translocate effector proteins into the host cells. The translocated effectors are crucial for bacterial infection and survival. The base of the T3SS transverses both bacterial membranes and contains an export apparatus that comprises five membrane proteins. Here, we study the export apparatus of enteropathogenic Escherichia coli (EPEC) and characterize its central component, called the EscR protein. We found that the third transmembrane domain (TMD) of EscR mediates strong self-oligomerization in an isolated genetic reporter system. Replacing this TMD sequence with an alternative hydrophobic sequence within the full-length protein resulted in a complete loss of function of the T3SS, further suggesting that the EscR TMD3 sequence has another functional role in addition to its role as a membrane anchor. Moreover, we found that an aspartic acid residue, located at the core of EscR TMD3, is important for the oligomerization propensity of TMD3 and that a point mutation of this residue within the full-length protein abolishes the T3SS activity and the ability of the bacteria to translocate effectors into host cells.

**IMPORTANCE** Many Gram-negative bacterial pathogens that cause life-threatening diseases employ a type III secretion system (T3SS) for their virulence. The T3SS comprises several proteins that assemble into a syringe-like structure dedicated to the injection of bacterial virulence factors into the host cells. Although many T3SS proteins are transmembrane proteins, our knowledge of these proteins is limited mostly to their soluble domains. In this study, we found that the third transmembrane domain (TMD) of EscR, a central protein of the T3SS in enteropathogenic E. coli, contributes to protein self-oligomerization. Moreover, we demonstrated that a single aspartic acid residue, located at the core of this TMD, is critical for the activity of the full-length protein and the function of the entire T3SS, possibly due to its involvement in mediating TMD-TMD interactions. Our findings should encourage the mapping of the entire interactome of the T3SS components, including interactions mediated through their TMDs.

## Abstract (precis)

Many Gram-negative bacterial pathogens that cause life-threatening diseases employ a type III secretion system (T3SS) for their virulence. The T3SS comprises several proteins that assemble into a syringe-like structure dedicated to the injection of bacterial virulence factors into the host cells. Although many T3SS proteins are transmembrane proteins, our knowledge of these proteins is limited mostly to their soluble domains. In this study, we found that the third transmembrane domain (TMD) of EscR, a central protein of the T3SS in enteropathogenic E. coli, contributes to protein self-oligomerization. Moreover, we demonstrated that a single aspartic acid residue, located at the core of this TMD, is critical for the activity of the full-length protein and the function of the entire T3SS, possibly due to its involvement in mediating TMD-TMD interactions. Our findings should encourage the mapping of the entire interactome of the T3SS components, including interactions mediated through their TMDs.

## INTRODUCTION

Many pathogenic bacteria utilize delivery machineries, such as the type III secretion system (T3SS), to inject effector proteins into eukaryotic host cells, where they subvert cellular mechanisms for the benefit of the bacteria. The T3SS forms a syringe-like protein complex that transverses the two bacterial membranes, bridges the extracellular space, and forms a pore in the eukaryotic host cell membrane (1–4). Translocated effectors support bacterial virulence as they manipulate various cellular processes to prolong bacterial survival and reduce cellular immune responses (5, 6). The T3SS apparatus is well conserved among T3SSs of different pathogens such as *Salmonella*, *Shigella*, *Yersinia*, and pathogenic Escherichia coli and shares significant similarities with components of the flagellar system (7, 8).

The T3SS of enteropathogenic E. coli (EPEC), the causative agent of pediatric diarrhea (6), forms an ~3.5-MDa complex comprising more than 20 different proteins (9). These proteins assemble into substructures that form, from inside to outside, cytoplasmic rings, an export apparatus, a basal body, an extracellular needle, and a pore-forming complex embedded within the host cell membrane, called a translocon (10, 11). Among these substructures, three (the export apparatus, the basal body, and the translocon) contain transmembrane proteins (reviewed in reference 10).

The export apparatus, found in the central part of the transport channel, is composed of five membrane proteins: EscR, EscS, EscT, EscU, and EscV. Null strains of single genes in the related murine pathogen Citrobacter rodentium were found to be nonvirulent as they were defective in their ability to secrete T3SS effectors and translocators, to attach to host cells, and to inject effectors into the host cell cytoplasm (9). All export apparatus proteins are predicted to contain multiple transmembrane domains (TMDs). Combined with the recent stoichiometry study of the *Salmonella* export apparatus that reported a 5:1:1:1:9 ratio for SpaP-SpaQ-SpaR-SpaS-InvA proteins (12), the estimated number of TMDs found in an export apparatus of a single T3SS complex is over 100. While the cytoplasmic domains of EscU and EscV, and their homologs in T3SSs of other pathogens, were structurally and functionally characterized (13–21), our knowledge regarding EscR, EscS, and EscT proteins as well as the TMDs of EscV and EscU is very limited.

In the past, the common dogma was that TM helices merely represent membrane anchors of soluble proteins that facilitate the folding of the proteins across the membrane environment. However, in the last two decades many studies have reported that such helices often have fundamental roles in the assembly and function of TM complexes (22–25). This role is often executed by the ability of TMDs to self- or hetero-oligomerize within the membrane, initiating or stabilizing protein oligomerization or complex formation (26–31). This essential role is further highlighted by the identification of mutations in TMDs that are associated with severe diseases, ranging from cancer to amyloidal diseases (32–36). The involvement of TMDs in protein interactions was recently bolstered by a study showing that proteins of the T3SS export apparatus of *Salmonella* pathogenicity island 1 (SPI-1) self- and heterointeract via amino acids localized within the TMDs of the proteins (37).

In this study, we focused on the EscR protein, which was suggested to be the first T3SS protein to localize to the bacterial inner membrane, self-oligomerize, and initiate the assembly of the T3SS complex by interacting with additional export apparatus proteins (37). We characterized the type III secretion and translocation activities of an EPEC *escR* null mutant as well as strains complemented with labeled versions of *escR*. We investigated the ability of each individual EscR TMD to support self-oligomerization using a genetic reporter system that converts the interaction of a specific TMD, within its natural membrane, into reporter gene expression. We found that the third TMD of EscR self-oligomerizes and is critical for the proper activity of the T3SS complex. Moreover, we demonstrated that an aspartic acid residue found at the core of TMD3 is critical for TMD-TMD interactions and is crucial for the ability of the bacteria to secrete and translocate effectors into host cells.

## RESULTS

### The *escR* gene is critical for T3SS activity and can be complemented by a labeled protein.

**FIG 1:**
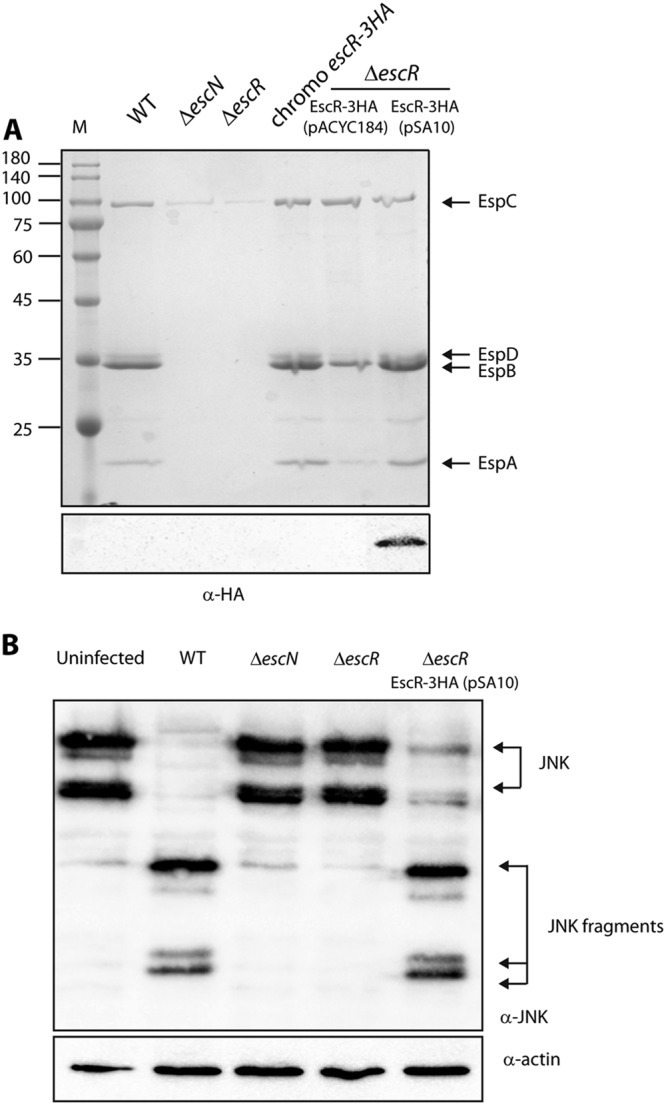
C-terminally labeled EscR can complement the Δ*escR* mutant. (A) Protein secretion profiles of EPEC strains grown under T3SS-inducing conditions: wild type (WT), Δ*escN* mutant (a T3SS ATPase mutant), Δ*escR* mutant, chromosomal *escR*-*3HA* strain, and Δ*escR* mutant complemented with EscR-3HA (encoded on either pACYC184 or pSA10). The secreted proteins were concentrated from supernatants of bacterial cultures and analyzed by SDS-PAGE and Coomassie blue staining. The T3SS-secreted translocators EspA, EspB, and EspD are indicated on the right of the gel. Also indicated is the location of EspC, which is not secreted by the T3SS. For the Δ*escN* and Δ*escR* strains, no T3SS activity was observed. Strains carrying the chromosomal *escR-3HA* or the plasmids encoding EscR-3HA showed proper T3SS activity. The expression of EscR-3HA was examined by analyzing the bacterial pellets by SDS-PAGE and Western blot analysis with an anti-HA antibody. EscR-3HA expression was detected only for EscR-3HA expressed from a high-copy-number plasmid, pSA10. Numbers at left are molecular masses in kilodaltons. (B) To examine whether pEscR-3HA can complement the Δ*escR* mutant in a host cell infection model, HeLa cells were infected with EPEC strains: WT, Δ*escN*, and Δ*escR* strains and Δ*escR* strain complemented with pEscR-3HA. After 3 h, cells were washed, and host cell proteins were extracted and subjected to Western blot analysis using anti-JNK and anti-actin (loading control) antibodies. JNK and its degradation fragments are indicated at the right of the gel. WT EPEC showed massive degradation of JNK compared to the uninfected sample and the samples infected with Δ*escN* or Δ*escR* mutant strains. The Δ*escR* strain complemented with pEscR-3HA showed a similar JNK degradation profile as WT EPEC, thus suggesting that pEscR-3HA is functional and therefore fully complements the Δ*escR* infection phenotype.

It was previously shown that the five T3SS export apparatus proteins of the EPEC-related murine pathogen C. rodentium are critical for T3SS activity, pedestal formation, and infection of host cells (9). In addition, EPEC strains carrying a single gene deletion of *escV* or *escU* were previously shown to completely abolish EPEC T3SS activity (21, 38). To examine whether the remaining export apparatus genes, *escR*, *escS*, and *escT*, are critical for EPEC T3SS activity, we generated single mutant EPEC strains and examined their T3SS activity. Wild-type (WT) EPEC, grown under T3SS-inducing conditions, secretes the type III secretion (T3S) translocators EspA, EspB, and EspD into the culture supernatant (Fig. 1A). We observed that the Δ*escR* EPEC strain, grown under similar conditions, secreted no translocators and displayed a secretion pattern similar to that of the Δ*escN* EPEC strain, deleted for the T3SS ATPase gene. Similar results were obtained for Δ*escS* and Δ*escT* null mutants (see Fig. S1 in the supplemental material), thus indicating that all export apparatus proteins are crucial for proper T3SS activity.

10.1128/mSphere.00162-18.2 FIG S1  All export apparatus genes are critical for T3SS activity. Protein secretion profiles of wild-type (WT), Δ*escR*, Δ*escS*, Δ*escT*, Δ*escU*, and Δ*escV* EPEC strains grown under T3SS-inducing conditions. The secreted proteins were concentrated from supernatants of bacterial cultures and analyzed by SDS-PAGE and Coomassie blue staining. The T3SS-secreted translocators EspA, EspB, and EspD are indicated on the right of the gel. Also indicated is the location of EspC, which is not secreted by the T3SS. T3SS activity was observed for the WT EPEC strain but not in any of the mutant strains. Download FIG S1, TIF file, 2.1 MB. Copyright © 2018 Tseytin et al. 2018 Tseytin et al. This content is distributed under the terms of the Creative Commons Attribution 4.0 International license.

To examine whether a labeled EscR can maintain T3SS activity, we labeled the chromosomal *escR* gene with a C-terminal triple-hemagglutinin (3HA) tag and examined its T3SS activity. We observed that the strain showed similar T3SS activity as WT EPEC, but no EscR-3HA expression was detected in the bacterial whole-cell lysates (Fig. 1A). This suggested that native expression levels of EscR are relatively low. To detect EscR expression, we cloned EscR-3HA on a low-copy-number plasmid (pACYC184) and a high-copy-number plasmid (pSA10) and transformed the plasmids into an Δ*escR* EPEC strain. The strains were grown under T3SS-inducing conditions and were shown to have complemented T3SS activity (Fig. 1A). However, EscR-3HA expression was detected only in the whole-cell lysates of the Δ*escR* strain transformed with EscR-3HA expressed from the high-copy-number plasmid but not from the low copy-number plasmid (Fig. 1A). These results suggested that the expression level of EscR is highly regulated.

To examine whether overexpression of EscR-3HA interferes with the activity of EPEC in infecting host cells, we examined the ability of the Δ*escR* strain carrying EscR-3HA to translocate effectors into the host cells. For this purpose, we infected HeLa cells with various EPEC strains (WT, Δ*escN*, Δ*escR*, and Δ*escR* complemented with pEscR-3HA) and examined the cleavage pattern of Jun N-terminal protein kinase (JNK), a host protein that is cleaved by a translocated EPEC effector, called NleD (39). WT EPEC showed an extensive degradation of JNK, in contrast to the uninfected sample and the samples infected with Δ*escN* or Δ*escR* mutant strains (Fig. 1B). The EPEC Δ*escR* strain complemented with EscR-3HA showed JNK degradation, indicating functional complementation by the vector-encoded, 3HA-labeled EscR protein (Fig. 1B).

### The role of EscR TMDs in the activity of the protein.

**FIG 2:**
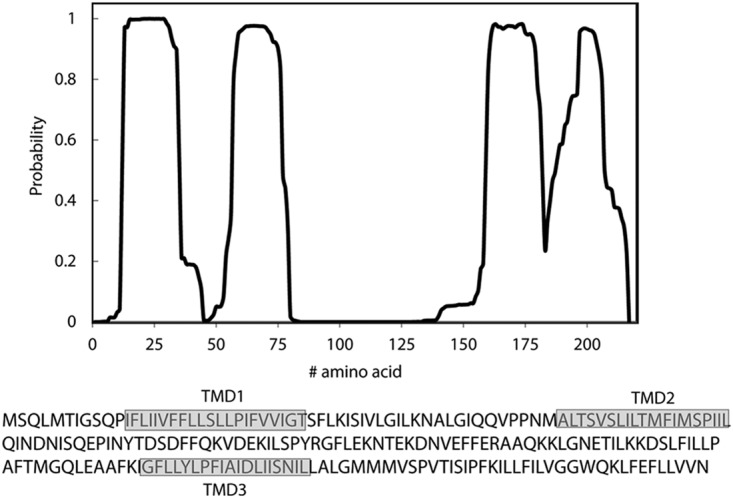
Prediction of EscR TMDs. Analysis of the EscR sequence for the probability of each amino acid to be localized within the membrane, using the prediction software TMHMM (78). Three clear TMDs (TMDs 1 to 3) were identified, and a fourth, shorter sequence was found to have a high probability to cross the membrane milieu. The sequences of the core TMDs are marked in gray boxes.

EscR, as well as the rest of the export apparatus proteins, is a membrane protein found at the center of the inner ring (40, 41). To predict the TMDs of EscR, we analyzed its sequence using TMD prediction software (TMHMM, TMPred, and SACS). Overall, three EscR regions were determined as having high probability to adopt a TMD orientation and an additional region with a medium-high probability was identified (Fig. 2). In this study, we decided to focus on the three strongly predicted TMD sequences, labeled TMD1, -2, and -3 (Fig. 2).

**FIG 3:**
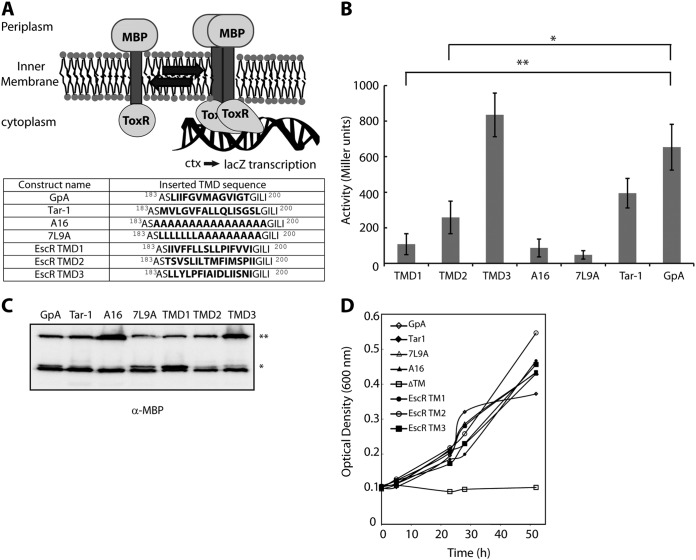
TMD3 of EscR promotes TMD self-oligomerization. (A) Schematic illustration of a ToxR assembly system. Oligomerization of the TMDs promotes the activation of the transcription activator ToxR, which binds the *ctx* promoter and initiates *lacZ* transcription. The TMD sequences that were inserted between the ToxR transcription activator and the maltose binding protein in the ToxR-TMD-MBP plasmid are presented. (B) FHK12 bacteria expressing a ToxR-TMD-MBP chimera were examined for LacZ activity. The activities of well-characterized dimerizing (GpA and Tar-1) and nondimerizing (A16 and 7L9A) TMDs are also shown. The oligomerization ability of the EscR TMD1 was low, TMD2 had moderate ability, and TMD3 showed very strong TMD oligomerization activity. Bars represent the average (+standard deviation) from at least three independent experiments. Statistical significance was determined by Student’s *t* test (**, *P* < 0.005; *, *P* < 0.01). (C) Samples of FHK12 cells containing the ToxR-TMD-MBP chimera proteins with different TMD sequences were lysed, separated, and immunoblotted using an anti-MBP antibody. The ToxR-TMD-MBP chimera protein (65 kDa) is marked with **, and the endogenous MBP (40 kDa) is marked with *. (D) Correct integration of the ToxR-TMD-MBP chimera proteins was tested by assessing their ability to functionally complement the *malE* deficiency of the PD28 bacterial strain. PD28 bacteria were transformed with plasmids expressing chimera proteins containing the EscR TMD1, TMD2, TMD3, GpA, Tar-1, 7L9A, or A16 or in the absence of a TMD (ΔTM) and were grown in a minimal medium containing maltose. As expected, the ΔTM negative control showed no growth, while all other constructs showed growth, indicating proper membrane integration.

TMDs have previously been shown to be involved in self- and hetero-oligomerization of proteins (29, 42–45). To assess the ability of single EscR TMDs to mediate self-oligomerization, we utilized the ToxR assembly system, which is designed to detect protein-protein interactions within the membrane of E. coli (Fig. 3A). We used the glycophorin A (GpA) TMD sequence as a positive control for strong homo-oligomerization (46–48). The GpA TMD contains a GxxxG motif, which is the most common and the best-characterized motif for interactions of transmembrane helices. The N-terminal TMD of the E. coli aspartate receptor (Tar-1) was used as a reference for moderate oligomerization, as this sequence contains a QxxS motif, which has about 50% self-oligomerization activity relative to the GpA TMD (49). Polyalanine (A16) and 7-leucine-9-alanine (7L9A) sequences were used as controls for nonoligomerizing sequences (50, 51). We observed a strong TMD self-oligomerization activity of EscR TMD3 compared to the activities of the GpA and Tar-1 TMDs, whereas the EscR TMD1 and TMD2 showed significantly lower oligomerization activities than GpA (Fig. 3B). As expected, the oligomerization of both background controls—A16 and 7L9A—was low (Fig. 3B). These findings suggested that the TMD3 of EscR might be involved in EscR self-oligomerization through TMD-TMD interactions. To exclude the possibility that the high self-oligomerization activity of EscR TMD3 was due to the high expression level of the chimera protein, we subjected the bacterial samples to SDS-PAGE and Western immunoblotting analysis with an anti-maltose binding protein (MBP) antibody. The chimera protein constructs showed comparable expression levels, with A16 and TMD3 constructs having a slightly higher expression level (Fig. 3C). Since the higher expression of the A16 construct did not result in high oligomerization activity, we concluded that the slightly higher expression of the TMD3 construct cannot explain, by itself, the strong oligomerization activity observed for this TMD sequence. To determine the correct integration of the ToxR-TMD-MBP chimera proteins into the inner membrane of E. coli, we employed the maltose complementation assay using PD28 bacterial strains carrying the ToxR-TMD-MBP constructs described in Fig. 3A (50). The PD28 strain is deleted for the *malE* gene, and therefore, it cannot produce endogenous MBP, which is involved in maltose catabolism and supports bacterial growth in minimal medium with maltose as the sole carbon source. Phenotype complementation (growth in maltose-only medium) will be observed only for PD28 strains that express the chimera protein ToxR-TMD-MBP and orient it correctly across the inner membrane, with MBP facing the periplasm. Using this system, we observed that all strains carrying the various ToxR-TMD-MBP constructs demonstrated similar growth rates (indicating proper membrane integration) while the negative control—a chimera protein deleted for its TMD (ΔTM)—showed no growth, as expected (Fig. 3D).

### Replacing the EscR TMD3 sequence with a hydrophobic sequence impairs the function of the protein.

**FIG 4:**
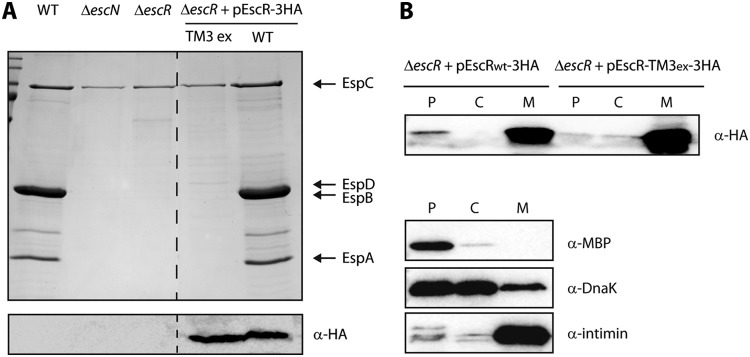
EscR TMD3 is critical for the T3SS activity. (A) Protein secretion profiles of WT, Δ*escN*, Δ*escR*, and Δ*escR* complemented with EscR_WT_-3HA or EscR-TM3_ex_-3HA EPEC strains grown under T3SS-inducing conditions. The secreted fractions were treated and analyzed as described in the Fig. 1A legend. Replacement of the TMD3 sequence with a 7L9A sequence completely abolished T3SS activity. The expression of EscR_WT_-3HA and EscR-TM3_ex_-3HA was examined by analyzing the bacterial pellets by SDS-PAGE and Western blot analysis with an anti-HA antibody. (B) The Δ*escR* EPEC strain carrying either EscR_WT_-3HA or EscR-TM3_ex_-3HA vector was grown under T3S-inducing conditions and was fractionated into periplasmic (P), cytoplasmic (C), and membrane (M) fractions. The samples were separated on an SDS-PAGE gel and analyzed by Western blotting using anti-HA antibody. To confirm correct bacterial fractionation, the Western blots were probed with anti-MBP (periplasmic marker), anti-DnaK (cytoplasmic marker), and anti-intimin (membrane marker) antibodies.

To determine whether the EscR TMD3 performs a role in the full-length protein, other than membrane anchoring, we constructed a mutant EscR protein lacking its TMD3 sequence. Since a ΔTMD3 EscR protein will likely have impaired localization or adopt an alternate protein folding compared to the native protein, we had to use an alternative design to study the function of TMD3. For this purpose, we constructed a TMD3-exchanged EscR protein, which had a WT sequence with a replacement of its native core TMD3 sequence (16 amino acids in length) by a hydrophobic sequence composed of seven sequential leucine residues followed by nine alanine residues (7L9A). The core 7L9A sequence, embedded between 5 hydrophobic amino acids of the original TMD, was previously shown to be sufficiently hydrophobic to support protein integration into the membrane, but it cannot support TMD-TMD interactions (51) (Figure 3B). To examine the biological effect of such a replacement, we transformed the TMD3-exchanged EscR (pEscR-TM3_ex_-3HA) into the *escR*-null strain (Δ*escR*) and examined its ability to complement the T3SS activity. We observed that while EscR-3HA complemented the T3SS activity of the Δ*escR* strain to a level similar to that of WT EPEC, the pEscR-TM3_ex_-3HA protein showed no T3SS activity (Fig. 4A). Comparable protein expression and similar cellular localization of the EscR_WT_-3HA protein and its TMD3-exchanged version were verified by Western immunoblotting with an anti-HA antibody (Fig. 4A [lower panel] and B, respectively). These findings indicate that the EscR TMD3 sequence is critical not only for targeting and orienting EscR across the membrane but also for the function of the protein, probably by mediating its correct folding, interaction, and/or assembly.

### The aspartic acid residue is critical for EscR TMD3 oligomerization.

**FIG 5:**
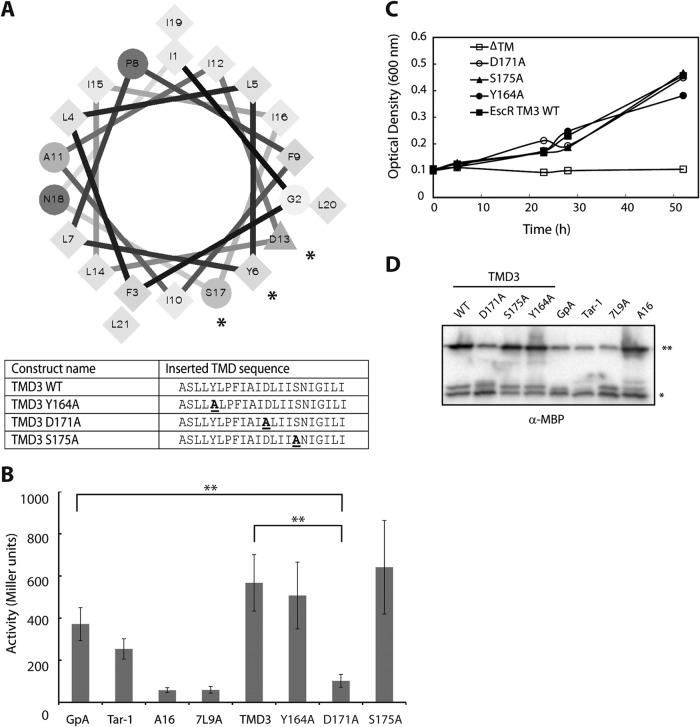
The aspartic acid residue found in the predicted EscR TMD3 sequence is critical for TMD self-oligomerization. (A) Helical wheel representation of the EscR TMD3 sequence demonstrating that three polar residues (Y164, D171, and S175) localized to one interface. The polar residues are marked with *. The EscR WT TMD3 sequence inserted between ToxR and MBP, as well as the point mutation sequences, is presented in a table. (B) LacZ activity of FHK12 bacteria expressing the ToxR-TMD-MBP chimeras. The activities of well-characterized dimerizing (GpA and Tar-1) and nondimerizing (A16 and 7L9A) TMDs are also shown. A major reduction in the oligomerization activity of EscR TMD3 was observed for the point mutation D171A, while the point mutations Y164A and S175A showed similar oligomerization activity as EscR TMD3 WT sequence. Bars represent the average (+standard deviation) from at least three independent experiments. Statistical significance was determined by Student’s *t* test (**, *P* < 0.005). (C) Correct integration of the ToxR-TMD-MBP chimera proteins was examined as described in the Fig. 3D legend. PD28 bacteria were transformed with plasmids expressing chimera proteins containing the EscR TMD3 WT sequence or the Y164A, S175A, or D171A mutation or in the absence of a TMD (ΔTM) and were grown in a minimal medium containing maltose. The ΔTM negative control showed no growth, whereas all the other constructs showed similar growth curves, indicating proper membrane integration. (D) Samples of FHK12 cells containing the ToxR-TMD-MBP chimera protein with WT EscR TMD3 and single mutations were lysed, separated, and immunoblotted using an anti-MBP antibody. The ToxR-TMD-MBP chimera protein (65 kDa) is marked with **, and the endogenous MBP (40 kDa) is marked with *.

A helical wheel presentation of EscR TMD3, which illustrates the location of individual amino acid residues within a sequence that adopts an alpha-helical structure, as expected for most TMDs, showed that 3 polar residues (Y164, D171, and S175) are located on the same helical interface of TMD3 (Fig. 5A). As polar and aromatic residues within the sequence of TMDs have been previously shown to be involved in TMD-TMD interactions (52–57), we examined whether these residues are critical for TMD self-oligomerization in EscR. For this purpose, we constructed three vectors expressing ToxR-TMD3-MBP chimera proteins with single point mutations (Y164A, D171A, and S175A; the exact sequences are shown in Fig. 5A) and examined their ability to mediate self-oligomerization. We observed that while the activity of Y164A and S175A mutants was rather similar to that of EscR TMD3 WT, the D171A mutant showed significantly reduced TMD-TMD interaction compared to EscR TMD3 WT or GpA TMD (Fig. 5B). This result suggested that the aspartic acid residue at position 171 is critical for TMD-TMD interactions mediated by TMD3. Correct localization of the ToxR-TMD-MBP chimera proteins was examined as described above, and all the ToxR-TMD-MBP chimera proteins were shown to be properly integrated within the membrane (Fig. 5C). The expression levels of ToxR-TMD-MBP were examined by subjecting bacterial samples to SDS-PAGE and Western immunoblotting analysis with an anti-MBP antibody. The expression level of the chimera proteins ToxR-TMD3-MBP of WT, Y164A, and S175A was higher than the GpA TMD (Fig. 5D). This high expression level might provide an explanation for the unusually high activity of these constructs relative to the GpA TMD control. Since the expression level of the ToxR-TMD3-MBP D171A mutant was similar to that of ToxR-GpA-MBP (Fig. 5D), we can exclude the possibility that the low oligomerization activity of the D171A mutant was due to low expression of the chimera protein.

### A single mutation at the EscR TMD3 abolishes the T3SS activity of EPEC and its ability to translocate effectors into host cells.

**FIG 6:**
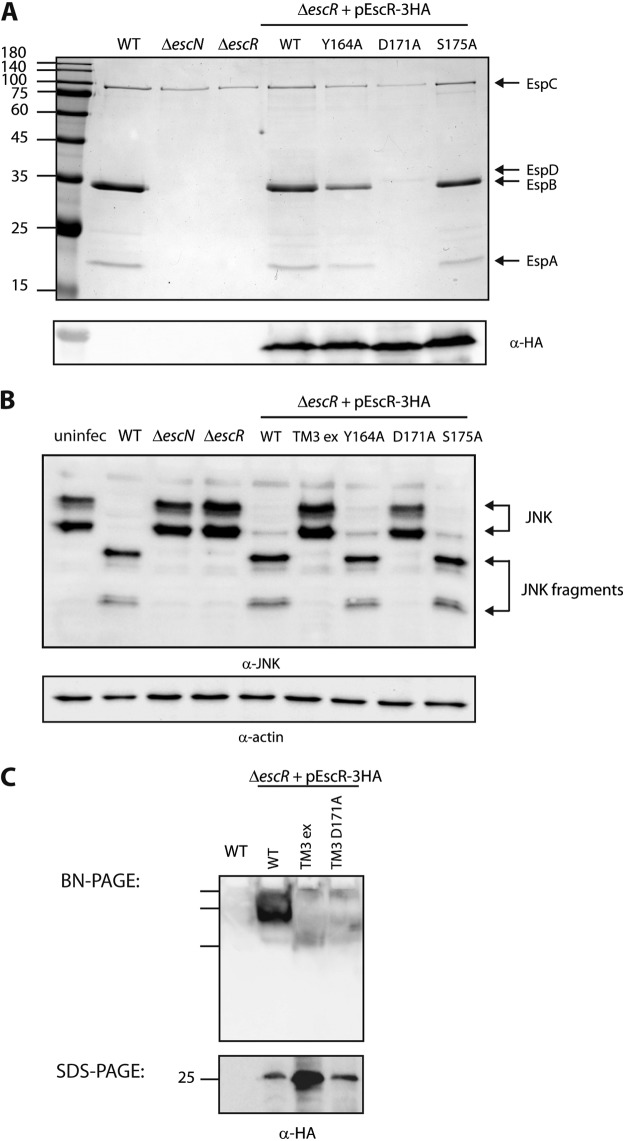
The aspartic acid residue found in the predicted EscR TMD3 sequence is critical for T3SS activity and the ability of the bacteria to infect host cells. (A) Protein secretion profiles of EPEC strains grown under T3SS-inducing conditions: WT, Δ*escN* and Δ*escR* strains, and Δ*escR* strain complemented with EscR_WT_-3HA, EscRY_164A_-3HA, EscRD_171A_-3HA, or EscRS_175A_-3HA. The secreted fractions were treated and analyzed as described in the Fig. 1A legend. A point mutation at position 171 of EscR TMD3 (D to A) abolished T3SS activity, while point mutations at position 164 or 175 (Y to A or S to A, respectively) demonstrated active T3SS. The expression of EscR-3HA variants was examined by analyzing the bacterial pellets by SDS-PAGE and Western blot analysis with an anti-HA antibody. Numbers at left are molecular masses in kilodaltons. (B) HeLa cells were infected with one of the following EPEC strains: WT, Δ*escN* or Δ*escR* strain, or Δ*escR* strain complemented with EscR_WT_-3HA, EscR-TM3_ex_-3HA, EscRY_164A_-3HA, EscRD_171A_-3HA, or EscRS_175A_-3HA. JNK and its degradation fragments are indicated at the right of the gel. WT EPEC showed massive degradation of JNK similarly to the Δ*escR* strain complemented with EscR_WT_-3HA, EscRY_164A_-3HA, or EscRS_175A_-3HA. However, Δ*escR* EPEC strains transformed with EscR-TM3_ex_-3HA or EscRD_171A_-3HA showed the same JNK pattern as the uninfected sample and the samples infected with Δ*escN* or Δ*escR* mutant strains. (C) Membrane protein extracts of WT EPEC and Δ*escR* mutant complemented with EscR_WT_-3HA, EscR-TM3_ex_-3HA, or EscRD_171A_-3HA were incubated in BN sample buffer and then subjected to BN-PAGE (upper panel) and SDS-PAGE (lower panel) and Western blot analysis using anti-HA antibody. (Upper panel) BN-PAGE analysis showed that EscR_WT_-3HA forms a high-molecular-weight protein complex that is absent from the EscR-TM3_ex_-3HA and the EscRD_171A_-3HA samples. We observed only three marker bands in the Western blot of BN-PAGE; therefore, we cannot estimate the size of the complex. (Lower panel) To confirm similar EscR expression levels among the samples, membrane protein extracts were analyzed by SDS-PAGE and Western blot analysis using anti-HA antibody. Similar protein expression levels were observed.

To examine the correlation between the oligomerization propensity, observed using the ToxR-TMD-MBP system, and the activity of the full-length EscR protein, we mutated the vector-expressed EscR-3HA to express the Y164A, D171A, or S175S mutant form and examined its ability to complement the T3SS activity of the Δ*escR* mutant. We found that the point mutation at position 171 abolished T3SS activity while point mutations of tyrosine, at position 164, or serine, at position 175, to alanine exhibited functional T3SS activities (Fig. 6A). To rule out expression defects of the EscR mutant form, we subjected whole-cell lysates to SDS-PAGE and Western blot analysis with an anti-HA antibody. We observed similar expression levels of EscR_WT_-3HA, EscRY_164A_-3HA, EscRD_171A_-3HA, and EscRS_175A_-3HA (Fig. 6A). Next, we examined the ability of the Δ*escR* strain carrying the mutant forms of EscR to properly translocate bacterial effectors into host cells. We infected HeLa cells with WT EPEC or Δ*escN* or Δ*escR* strains carrying either pEscR_WT_-3HA or pEscR-TM3_ex_-3HA or the plasmids carrying the single EscR mutants (Y164A, D171A, or S175A). As previously reported, WT EPEC showed extensive degradation of JNK, compared to the uninfected sample and the samples infected with the Δ*escN* or Δ*escR* mutant strain (Fig. 1B and 6B). EPEC Δ*escR* complemented with EscR_WT_-3HA or with EscR mutated at position 164 or 175 showed similar JNK degradation profiles as observed for WT EPEC, while TMD3-exchanged EscR and EscR mutated at position 171 were not able to translocate effectors into HeLa cells (Fig. 6B). These results suggest that EscR TMD3 and specifically the aspartic acid residue at position 171 are critical for the proper activity of the protein.

To determine whether EscR can form large oligomers, similar to its functional homologs, and examine whether this is affected by the replacement of EscR TMD3 sequence or a D171A mutation, we extracted membrane proteins of WT EPEC and the Δ*escR* strain complemented with EscR_WT_-3HA, EscR-TM3_ex_-3HA, or EscRD_171A_-3HA. The samples were analyzed by blue native PAGE (BN-PAGE) and Western immunoblotting with an anti-HA antibody. This method was used since repeated attempts to purify the EscR protein in order to examine the oligomerization propensity of the isolated protein were unsuccessful. The membrane protein extract of the Δ*escR* strain carrying the pEscR_WT_-3HA vector contained large complexes, which migrated slowly in the BN-PAGE gel, thus supporting EscR oligomerizing behavior (Fig. 6C, upper panel). Since only three marker bands appeared in the Western blot assay of the BN-PAGE (their locations are marked), we were not able to estimate the size of the complexes. The membrane protein extracts of the Δ*escR* strain carrying pEscR-TM3_ex_-3HA or pEscR_D171A_-3HA showed a relatively smeared HA signal that lacked the appearance of a large protein complex as observed for the EscR_WT_-3HA sample (Fig. 6C, upper panel). Examination of the membrane protein extracts of the Δ*escR* strain carrying pEscR_Y164A_-3HA showed formation of high-molecular-weight complexes similar to that formed by pEscR_WT_-3HA, while the membrane protein extract of the Δ*escR* strain carrying pEscR_S171A_-3HA showed an intermediate phenotype (see Fig. S2 in the supplemental material). These results suggested that the replacement of the TMD3 sequence by an alternative hydrophobic domain or a point mutation at the conserved aspartic acid residue altered the ability of the EscR protein to oligomerize. To confirm that the altered running pattern of EscR-TM3_ex_-3HA and EscRD_171A_-3HA was not due to lower expression of the mutant forms than of the WT protein, we loaded membrane protein extracts onto an SDS-PAGE gel and analyzed them by Western blotting using anti-HA antibody. We observed similar expression levels of EscR_WT_-3HA and EscRD_171A_-3HA, while the EscR-TM3_ex_-3HA protein showed a higher expression level (Fig. 6C, lower panel). Overall, these results confirmed that replacement of the TMD3 sequence or mutating D171 to alanine reduces the ability of EscR to self- or hetero-oligomerize.

10.1128/mSphere.00162-18.3 FIG S2  Oligomer formation of EscR point mutations. Membrane protein extracts of WT EPEC and Δ*escR* strain complemented with EscR_WT_-3HA, EscR-TM3_ex_-3HA, EscRY_164A_-3HA, EscRD_171A_-3HA, or EscRS_175A_-3HA were incubated in BN sample buffer and then subjected to BN-PAGE (3 to 8% gradient gel; upper panel) and SDS-PAGE (lower panel) and Western blot analysis using anti-HA antibody. (Upper panel) BN-PAGE analysis showed that EscR_WT_-3HA and EscRY_164A_-3HA form a high-molecular-weight protein complex that is absent from the EscR-TM3_ex_-3HA and the EscRD_171A_-3HA samples. EPEC Δ*escR* transformed with pEscR_S175A_-3HA showed an intermediate phenotype. (Lower panel) To confirm similar EscR expression levels among the samples, membrane protein extracts were analyzed by SDS-PAGE and Western blot analysis using anti-HA antibody. Similar protein expression levels were observed. Download FIG S2, TIF file, 0.5 MB. Copyright © 2018 Tseytin et al. 2018 Tseytin et al. This content is distributed under the terms of the Creative Commons Attribution 4.0 International license.

**FIG 7:**
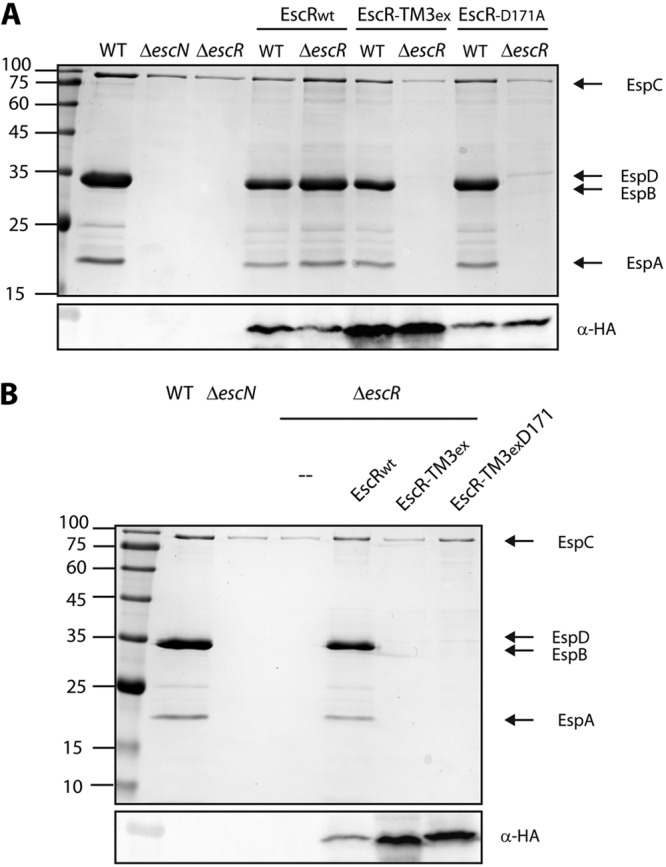
Dysfunctional versions of EscR have no dominant negative effect and cannot be rescued by insertion of an aspartic acid residue into the TMD sequence. (A) Protein secretion profiles of WT and Δ*escR* EPEC strains transformed with EscR_WT_-3HA, EscR-TM3_ex_-3HA, or EscRD_171A_-3HA grown under T3SS-inducing conditions. The secreted fractions were treated and analyzed as described in the Fig. 1A legend. The expression of EscR-3HA variants was examined by analyzing the bacterial pellets by SDS-PAGE and Western blot analysis with an anti-HA antibody. Numbers at left are molecular masses in kilodaltons. (B) Protein secretion profiles of Δ*escR* EPEC transformed with EscR_WT_-3HA, EscR-TM3_ex_-3HA, or EscRTM3_ex_D171-3HA grown under T3SS-inducing conditions. The secreted fractions were treated and analyzed as described in the Fig. 1A legend. The expression of EscR-3HA variants was examined by analyzing the bacterial pellets by SDS-PAGE and Western blot analysis with an anti-HA antibody.

To determine whether nonfunctional EscR constructs (which are unable to complement Δ*escR* strain T3SS activity) disrupt the T3SS of WT EPEC, we transformed pEscR_WT_-3HA, pEscR-TM3_ex_-3HA, or pEscR_D171A_-3HA into the WT EPEC strain and examined the strains’ T3SS activity. We observed that pEscR-TM3_ex_-3HA and pEscR_D171A_-3HA have no dominant negative effect when expressed in the WT EPEC background (Fig. 7A). These results suggested either that the dysfunctional copies of EscR are unable to assemble into the EscR complex due to their mutations or that not all EscR copies within the T3SS complex are required to be functional. To determine whether the aspartic acid residue at position 171 is sufficient to support EscR oligomerization and activity, we mutated the pEscR-TM3_ex_-3HA construct to contain an aspartic acid at position 171. The resulting construct, pEscR-TM3_ex_D171-3HA, contained a nonfunctional sequence (7L9A) instead of the native TMD3 sequence with a point mutation, A171D. We transformed the plasmid into the Δ*escR* strain and examined its ability to restore the T3SS activity. We found that the pEscR-TM3_ex_D171-3HA was unable to rescue the T3SS activity, thus suggesting that inserting an aspartic acid residue at position 171 is not sufficient to support proper protein oligomerization and function and that the surrounding TMD sequence contributes to the overall activity (Fig. 7B). Whole-cell lysates were subjected to SDS-PAGE and Western blot analysis with an anti-HA antibody and confirmed expression of all EscR versions (Fig. 7B).

## DISCUSSION

The export apparatus comprises five membrane proteins, which are highly conserved among T3SSs of different pathogens. The export apparatus was suggested to assemble within the bacterial inner membrane, during the initial steps of the T3SS formation, and to be involved in substrate secretion regulation. While considerable information is available for the EscU and EscV components of the export apparatus, not much is reported for the EscR, EscS, and EscT proteins. In addition, although all export apparatus components are transmembrane proteins and the recent estimation suggested that over 100 TMDs are found in the inner membrane of a single T3SS complex (12), our knowledge about these proteins is limited mostly to their soluble domains. In this study, we focused mostly on the EscR protein and characterized the role of its TMDs in the activity of the protein.

Previous studies indicated that the EscR homologs form homo-oligomers; FliP, of *Salmonella* flagella, forms a homohexamer (58, 59), and SpaP of the *Salmonella* T3SS forms a homopentamer (37). Based on these results and the growing number of reports regarding the involvement of TMDs in mediating self-oligomerization within the membrane milieu (23, 29, 31, 45, 60, 61), we examined whether EscR TMDs mediate homo-oligomerization. Using the ToxR system, we identified that the third TMD of EscR (residues 159 to 179 of the EscR sequence) exhibits a strong self-oligomerization activity (Fig. 3B). The TMD3 self-oligomerization demonstrated a similar oligomerization level as the well-characterized GpA TMD. However, while the GpA TMD contains a GxxxG motif, a similar motif was not detected within the EscR TMD3 sequence. To examine whether polar/aromatic residues, which were previously reported to be involved in TMD-TMD interactions (23, 49, 55, 62–64), drive EscR TMD3 oligomerization, we mutated a tyrosine residue at position 164 (Y_164_), an aspartic acid residue at position 171 (D_171_), and a serine residue at position 175 (S_175_) to alanine and examined the effect of these single mutations on TMD3 oligomerization. We observed a significant reduction of oligomerization only for the D171A mutation in the EscR TMD3, thus suggesting that this amino acid residue is critical for the TMD self-interaction.

To examine whether the TMD3 is also critical for the activity of the full-length protein, we replaced TMD3 of EscR with a 7L9A sequence and examined whether this EscR TMD3-exchanged form can complement the T3SS activity of an *escR* mutant strain. Interestingly, we found that replacing the TMD3 sequence with an alternative hydrophobic sequence resulted in a completely nonfunctional T3SS (Fig. 4A). Moreover, this strain was unable to infect HeLa cells (Fig. 6B). These results suggested that the EscR TMD3 sequence has a functional role within the full-length protein, in addition to its role as a membrane anchor. Moreover, a point mutation of the aspartic acid residue at position 171 to alanine, which showed low oligomerization activity within the ToxR-MBP system, has shown an inability to complement the T3SS activity of an Δ*escR* mutant strain as well as its ability to infect HeLa cells. Protein sequence alignment of EscR with its homologs (FliP of the E. coli flagella, FliP of *Salmonella* flagella, SpaP of *Salmonella* SPI-1 T3SS, Spa24 of the *Shigella* T3SS, YscR of the *Yersinia* T3SS, and SsaR of *Salmonella* SPI-2 T3SS) showed high similarity between the proteins and full conservation of the aspartic acid residue at position 171 of EscR (see Fig. S3 in the supplemental material). Such conservation correlates well with our experimental data that this aspartic acid residue within the third TMD of EscR is critical for the activity of the protein. Consistent with our results, a recent study of the EscR homolog in *Salmonella*, SpaP, showed that a mutation of the aspartic acid residue at position 173 (equivalent to D_171_ in EscR) to the amber codon (UAG), which encodes an unnatural amino acid (*para*-benzophenylalanine) (65), resulted in a malfunctioning T3SS (37). Moreover, the authors reported that this aspartic acid residue is involved in SpaP self-interactions.

10.1128/mSphere.00162-18.4 FIG S3  Sequence alignment of export apparatus proteins. A standard protein BLAST alignment is presented by ClustalW (8) for FliP of E. coli flagella (P0AC05), FliP of *Salmonella* flagella (P54700), SpaP of *Salmonella* SPI-1 T3SS (P40700), SsaR of *Salmonella* SPI-2 T3SS (A0A0M0Q7P7), Spa24 of *Shigella* T3SS (P0A1L3), YscR of *Yersinia* T3SS (P69980), and EscR of EPEC T3SS (B7UMC1). High levels of conservation were observed among the proteins, including a 100% conservation of the aspartic acid residue at position 171 of EscR of EPEC T3SS. Download FIG S3, TIF file, 0.6 MB. Copyright © 2018 Tseytin et al. 2018 Tseytin et al. This content is distributed under the terms of the Creative Commons Attribution 4.0 International license.

To establish that EscR forms, similarly to its homologs, homo-oligomers, we extracted total membrane proteins from an EPEC Δ*escR* strain expressing EscR_WT_-3HA and analyzed it by BN-PAGE. Western blot analysis against the HA tag revealed that EscR forms a high-molecular-weight complex that migrates slowly in the gel. Since the sample is of complete membrane protein extraction, we cannot determine whether the high-molecular-weight band corresponds to homo-oligomers of EscR_WT_-3HA or to hetero-oligomers of EscR with additional bacterial proteins. Our attempts to purify EscR, using several tags and conditions, to better characterize the EscR-containing oligomers were unsuccessful due to low expression levels of the protein and its strong hydrophobic nature (data not shown). BN-PAGE analysis of membrane extracts of the Δ*escR* strain expressing either EscR-TM3_ex_-3HA or EscRD_171A_-3HA obtained a relatively smeared HA signal that lacked the high-molecular-weight complex that appeared in the EscR_WT_-3HA sample (Fig. 6C). These results suggested that the third TMD of EscR is involved in the oligomerization of the full-length protein and that, similarly to the isolated ToxR system, the D_171_ residue is critical for this oligomerization. This information explains the value of having a polar amino acid residue within the membrane environment despite its thermodynamic cost.

In conclusion, the present study demonstrates that the third TMD of EscR does not serve merely as a membrane anchor, as it cannot be replaced by an alternative hydrophobic sequence. Rather, this TMD sequence is involved in mediating EscR self-interactions, which was previously suggested to be the initial oligomerization process during the T3SS assembly (37). Following the formation of the EscR oligomer, hetero-oligomerization with additional export apparatus components can be induced to allow the stabilization of the export apparatus complex. Moreover, our findings establish that the polar aspartic acid residue at position 171 is critical for the activity and function of the full-length protein, possibly due to its involvement in TMD-TMD interaction. Our results, together with previous studies (37, 66, 67) that have reported the role of TMDs of T3SS components in supporting homo- and hetero-interactions as well as their involvement in the activity of the T3SS, should encourage further investigation in mapping the entire interactome of the T3SS components, including interactions that are mediated through the proteins’ TMDs.

## MATERIALS AND METHODS

The sequences of primers designed and used in this study are given in Table 1. Strains and plasmids used in this study are listed in Table 2.

**TABLE 1:** Sequences of primers designed and used in this study

Construct and primer designation	Primer sequence (restriction site)^a^
*escR* deletion mutant	
ESCR-01F	GGAGCTCATACCAGAGGATAAGTTACAGG (SacI)
ESCR-01R	CGCTAGCTATTGGCTGTGAGCCAATGGTC (NheI)
ESCR-02F	CGCTAGCTTCATTCTTGTTGGAGGCTGGC (NheI)
ESCR-02R	GGGTACCTGATATGCGTTCAGTCCTGTG (KpnI)
*escS* deletion mutant	
ESCS-01F	GGAGCTCATACCAGAGGATAAGTTACAGG (SacI)
ESCS-01R	CGCTAGCTTGAACAAAATATCCAGTATCC (NheI)
ESCS-02F	GGCTAGCGAAATGATTCCGAAGGTGAACG (NheI)
ESCS-02R	GGGTACCTGATATGCGTTCAGTCCTGTG (KpnI)
*escT* deletion mutant	
ESCT-01F	GGAGCTCATACCAGAGGATAAGTTACAGG (SacI)
ESCT-01R	CGCTAGCAAATGATGATACTATGACCGTC (NheI)
ESCT-02F	CGCTAGCACGGCAAATATTCATTCTGAC (NheI)
ESCT-02R	GGGTACCTGATATGCGTTCAGTCCTGTG (KpnI)
EscR-3HA cloning in pSA10	
EscR-3HA-F	TTTCACACAGGAAACAGATGTCTCAATTAATGACCATTGGCTCAC
EscR-3HA-R1	AGCGTAATCTGGAACATCGTATGGGTAATTCACCACCAACAGAAATTCG
EscR-3HA-R2	GATCCCCGGGAATTTCAAGCGTAATCTGGAACATCGTATGGGTAAGCGTAATCTGG
pSA10_Gib_F	AATTCCCGGGGATCCGTCG
pSA10_Gib_R	CTGTTTCCTGTGTGAAATTGTTATCCG
EscR-3HA cloning in pACYC184	
EscR-3HA-184_F	CCAGGATGAATAAAATTTAAAAATGTCTCAATTAATGACCATTG
EscR-3HA-184_R	CAAGGGCATCGGTCGACTCCCCGGGAATTTCAAGC
pACYC_Gib_F	GTCGACCGATGCCCTTG
pACYC_Gib_R	TTTTAAATTTTATTCATCCTGGTGGTTG
Labeling chromosomal EscR with 3HA	
EscR-3HA-Chr-F	ATGTCTCAATTAATGACCATTGGC
EscR-3HA-Chr-R	GGCTGCAGGTCGACGGATCCCCGGGAATTTCAAGCG
ESCR-03F	CCAAGCTTCTTCTAGAGGTACCTTGACATTCAATGCCCTG
ESCR-03R	GGTCATTAATTGAGACATATCATC
ESCR-04F	TCCGTCGACCTGCAGCCGATGGATACTGGATATTTTGTTC
ESCR-04R	AGCTCGATATCGCATGCGTCTATTGTCGTATTAACTAACCC
pRE112_Gib_F	GCATGCGATATCGAGCTC
pRE112_Gib_R	GGTACCTCTAGAAGAAGCTTG
TMD3-ex EscR	
7L9A-F	CTGTTGCTACTCTTACTCCTTGCGGCCGCAGCGGCTGCAGCGGCAGCC
7L9A-R	GGCTGCCGCTGCAGCCGCTGCGGCCGCAAGGAGTAAGAGTAGCAACAG
EscR_7L9A_F	CATCATACCCAAGGCCAATAAGGCTGCCGCTGCAGCCGCTGCGGC
EscR_7L9A_R	GCTGCATTCAAGATAGGTTTTCTGTTGCTACTCTTACTCCTTGCGGCC
EscR_Gib_F	CGGATAACAATTTCACACAGGAAACAGATGTCTCAATTAATG
EscR_Gib161_R	GAGTAAGAGTAGCAACAGAAAACCTATCTTGAATGCAGC
EscR_7L9A_Gib_R	CATCATACCCAAGGCCAATAAGGCTGCCGCTGCAGCCGC
pSA10_EscR_TM3_7L9A_F	TTATTGGCCTTGGGTATGATGATGGTATCG
pEscR-TM3_ex_D171-3HA	
L171D_F	CTCTTACTCCTTGCGGCCGACGCGGCTGCAGCGGCAGCC
L171D_R	GGCTGCCGCTGCAGCCGCGTCGGCCGCAAGGAGTAAGAG
TMD3 point mutants	
Y164F (ToxR)	GCTAGCTTGCTCGCTTTACCCTTTATTGCG
Y164R (ToxR)	CGCAATAAAGGGTAAAGCGAGCAAGCTAGCTCG
Y164F (EscR)	CAAGATAGGTTTTTTGCTCGCTTTACCCTTTATTGCG
Y164R (EscR)	CGCAATAAAGGGTAAAGCGAGCAAAAAACCTATCTTG
D171F	CCCTTTATTGCGATAGCTTTGATCATTTCCAATATC
D171R	GATATTGGAAATGATCAAAGCTATCGCAATAAAGGG
S175F (ToxR)	GATTTGATCATTGCTAATATCTTATTG
S175R (ToxR)	CAATAAGATATTAGCAATGATCAAATC
S175F (EscR)	CGATAGATTTGATCATTGCCAATATCTTATTGGCCTTGG
S175R (EscR)	CCAAGGCCAATAAGATATTGGCAATGATCAAATCTATCG
ToxR-TMD1-MBP	
EscR_TMD1_F	CTAGCATTATTGTATTTTTTCTGTTATCATTACTGCCAATATTTGTTGTTATTGG
EscR_TMD1_R	GATCCCAATAACAACAAATATTGGCAGTAATGATAACAGAAAAAATACAATAATG
ToxR-TMD2-MBP	
EscR_TMD2_F	CTAGCACATCAGTGTCTTTGATACTGACAATGTTTATTATGTCTCCGATAATAGG
EscR_TMD2_R	GATCCCTATTATCGGAGACATAATAAACATTGTCAGTATCAAAGACACTGATGTG
ToxR-TMD3-MBP	
EscR_TMD3_F	CTAGCTTGCTCTATTTACCCTTTATTGCGATAGATTTGATCATTTCCAATATCGG
EscR_TMD3_R	GATCCCGATATTGGAAATGATCAAATCTATCGCAATAAAGGGTAAATAGAGCAAG

a Restriction sites are underlined in primer sequences.

**TABLE 2:** Strains and plasmids used in this study

Strain or plasmid	Description	Reference
Strains		
Wild-type EPEC	EPEC strain E2348/69, streptomycin resistant	68
EPEC Δ*escR*	Nonpolar deletion of *escR*	This study
EPEC Δ*escS*	Nonpolar deletion of *escS*	This study
EPEC Δ*escT*	Nonpolar deletion of *escT*	This study
EPEC Δ*escN*	Nonpolar deletion of *escN*	38
EPEC Δ*escV*	Nonpolar deletion of *escV*	38
EPEC Δ*escU*	Nonpolar deletion of *escU*	21
E. coli DH10B	For plasmid handling	79
E. coli FHK12	E. coli strain in which *ctx* promoter was fused to *lacZ* gene	72
E. coli PD28	*malE-*deficient E. coli strain	77
E. coli SM10λ*pir*	Conjugating bacteria	80
EPEC *escR-3HA*	3HA C-terminally tagged chromosomal *escR*	This study
Plasmids		
pEscR_WT_-3HA (pACYC184)	3HA C-terminally tagged EscR in pACYC184	This study
pEscR_WT_-3HA (pSA10)	3HA C-terminally tagged EscR in pSA10	This study
pEscR-TM3_ex_-3HA (pSA10)	3HA C-terminally tagged EscR with 7L9A sequence instead of original TMD3 in pSA10	This study
pEscR-TM3_ex_D171-3HA (pSA10)	3HA C-terminally tagged EscR with 7L9A sequence instead of original TMD3 with aspartic acid in position 171	This study
EscRY_164A_-3HA (pSA10)	3HA C-terminally tagged EscR with Y164A mutation in pSA10	This study
EscRD_171A_-3HA (pSA10)	3HA C-terminally tagged EscR with D171A mutation in pSA10	This study
EscRS_175A_-3HA (pSA10)	3HA C-terminally tagged EscR with S175A mutation in pSA10	This study
ToxR-GpA-MBP	GpA TMD sequence inserted between ToxR and MBP	50
ToxR-Tar-1-MBP	N-terminal TMD of E. coli Tar sequenc inserted between ToxR and MBP	49
ToxR-A_16_-MBP	Sequence of 16 alanine residues inserted between ToxR and MBP	50
ToxR-7L9A-MBP	Sequence of 7 leucine residues and 9 alanine residues inserted between ToxR and MBP	51
ToxR-TMD1-MBP	First TMD sequence of EscR inserted between ToxR and MBP	This study
ToxR-TMD2-MBP	Second TMD sequence of EscR inserted between ToxR and MBP	This study
ToxR-TMD3-MBP	Third TMD sequence of EscR inserted between ToxR and MBP	This study
ToxR-TMD3_Y164A_-MBP	Third TMD sequence of EscR with Y164A mutation inserted between ToxR and MBP	This study
ToxR-TMD3_D171A_-MBP	Third TMD sequence of EscR with D171A mutation inserted between ToxR and MBP	This study
ToxR-TMD3_S175A_-MBP	Third TMD sequence of EscR with S175A mutation inserted between ToxR and MBP	This study
pACYC184	Cloning vector, Cm^r^ Tc^r^	81
pSA10	pKK177-3 derivative containing *lacI*^q^	82
pRE112	Suicide vector for allelic exchange, Cm^r^	83

### Bacterial strains.

The wild-type EPEC O127:H6 strain E2348/69 (a streptomycin-resistant strain) was used to assess the activity of the T3SS (68), and the E. coli DH10B strain was used for plasmid handling (Table 2). All strains were grown at 37°C in Luria-Bertani (LB) broth (Sigma) supplemented with the appropriate antibiotics. Antibiotics were used at the following concentrations: 50 µg/ml streptomycin, 50 µg/ml ampicillin, and 30 µg/ml chloramphenicol.

### Construction of null mutants and plasmids used in this study.

The details of the cloning processes are described in Text S1 in the supplemental material.

10.1128/mSphere.00162-18.1 TEXT S1  Detailed description of the construction of single null mutants and all plasmids used in this study and the membrane protein extraction method. Download TEXT S1, DOCX file, 0.04 MB. Copyright © 2018 Tseytin et al. 2018 Tseytin et al. This content is distributed under the terms of the Creative Commons Attribution 4.0 International license.

### Secretion assay.

To determine T3SS activity, EPEC strains were grown overnight in LB broth supplemented with the appropriate antibiotics in a shaker at 37°C. The cultures were diluted 1:50 into Dulbecco’s modified Eagle’s medium (DMEM; Biological Industries), preheated overnight in a CO_2_ tissue culture incubator, and grown statically supplemented with the appropriate antibiotics for 6 h in a tissue culture incubator (with 5% CO_2_) to an optical density at 600 nm (OD_600_) of 0.7. The cultures were then centrifuged at 14,000 × *g* for 5 min to remove the bacteria, the pellet was dissolved in the SDS-PAGE sample buffer, and the supernatant was collected and then filtered through a 0.22-µm filter (Millipore). The supernatant was then precipitated with 10% (vol/vol) trichloroacetic acid overnight at 4°C to concentrate proteins secreted into the culture medium. The volume of the supernatants was normalized to the bacterial culture OD_600_ to ensure equal loading of the samples. The samples were then centrifuged at 14,000 × *g* for 30 min at 4°C, the precipitates of the secreted proteins were dissolved in the SDS-PAGE sample buffer, and the residual trichloroacetic acid was neutralized with saturated Tris. The proteins were analyzed on SDS-12% PAGE gels and stained with Coomassie blue.

### Translocation activity.

To determine the ability of EPEC strains to translocate effectors into host cells, we performed translocation assays as previously described (39). Briefly, HeLa cells were infected for 3 h with EPEC strains (WT, Δ*escN*, Δ*escR*, and Δ*escR* complemented with EscR_WT_-3HA, pEscR-TM3_ex_-3HA, pEscR_Y64A_-3HA, pEscR_D171A_-3HA, or pEscR_S175A_-3HA) that were preinduced for 3 h for T3SS activity in preheated DMEM, statically, in a CO_2_ tissue culture incubator. HeLa cells were then washed with phosphate-buffered saline (PBS), collected, and lysed with radioimmunoprecipitation assay (RIPA) buffer. The samples were centrifuged at maximum speed for 5 min to remove unlysed cells, and the supernatants were collected, mixed with SDS-PAGE sample buffer, and subjected to Western blot analysis with anti-JNK and anti-actin antibodies (loading control). An uninfected sample and the Δ*escN* mutant strain-infected sample were used as negative controls.

### Immunoblotting.

Samples were subjected to SDS-PAGE and transferred to nitrocellulose membranes (pore size, 0.45 µm; Bio-Rad) or polyvinylidene difluoride (PVDF) (Mercury; Millipore). The blots were blocked for 1 h in 5% (wt/vol) skim milk-PBST (0.1% Tween in phosphate-buffered saline), incubated with the primary antibody (diluted in 5% skim milk-PBST for 1 h at room temperature, unless indicated otherwise) and then with the secondary antibody (diluted in 5% skim milk-PBST for 1 h at room temperature), and detected with the enhanced chemiluminescence (ECL) reagents (Biological Industries). The following primary antibodies were used: mouse anti-HA (Abcam), diluted 1:1,000; rabbit anti-MBP (Thermo Fisher Scientific), diluted 1:1,000; mouse anti-DnaK (Abcam); rabbit anti-intimin, diluted 1:2,000; mouse anti-JNK (BD Pharmingen), diluted 1:1,000 in Tris-buffered saline (TBS); and mouse anti-actin (MPBio), diluted 1:10,000. The following secondary antibodies were used: horseradish peroxidase-conjugated goat anti-mouse antibody (Abcam), diluted 1:5,000, and horseradish peroxidase-conjugated goat anti-rabbit antibody (Abcam), diluted 1:5,000.

### Detection of the homo-oligomerization of TMDs within the membrane.

The ToxR transcription activator can be successfully used to assess protein-protein interactions within the E. coli membrane (69). DNA cassettes, encoding single TMDs of EscR (TMD1, TMD2, and TMD3), E. coli aspartate receptor N-terminal TMD (Tar-1), glycophorin A (GpA) TMD, 16-alanine backbone (A16), 7-leucine-9-alanine backbone (7L9A), and no TMD (ΔTM), were grafted between the cytoplasmic domain of the ToxR transcription activator protein (an oligomerization-dependent transcriptional activator) and the periplasmic moiety of the maltose binding protein (MBP). The presence of the MBP moiety directs the localization of the chimera protein to the periplasm (70, 71) and, therefore, assists the TMD to become embedded within the inner membrane. In the assay, the ToxR-TMD-MBP plasmids, containing different TMDs, were transformed into E. coli FHK12 cells (72), which contain a reporter gene, coding for β-galactosidase, under the control of the *ctx* promoter. Oligomerization of the investigated TMD results in association of the ToxR transcription activator, which only then becomes active and can bind the *ctx* promoter to initiate transcription of a downstream reporter gene, *lacZ* (69, 73). Quantification of oligomerization is performed by calculating the activity of β-galactosidase, namely, by measuring the levels of a yellow color (OD_405_) associated with the cleavage product of the β-galactosidase substrate *o-*nitrophenylgalactose (ONPG) (23, 50, 51). Monitoring the activity of β-galactosidase for 20 min, at intervals of 30 s, yields the *V*_max_ of the reaction and is presented as Miller units when normalized to the original cell content (measured at OD_600_). We used the GpA TMD sequence as a positive control for strong homo-oligomerization (46–48), the N-terminal TMD of the E. coli aspartate receptor (Tar-1) as a reference for moderate oligomerization (49), and the A16 and 7L9A sequences as controls for nonoligomerizing sequences (51, 74–76).

### Maltose complementation assay.

Membrane insertion and correct orientation of the chimera proteins were examined as described previously (50). Briefly, PD28 cells (a *malE-*deficient E. coli strain [77]) transformed with the different ToxR-TMD-MBP plasmids were cultured overnight. The overnight cultures were washed twice with PBS and then used to inoculate M9 minimal medium supplemented with 0.4% maltose and chloramphenicol. Bacterial growth was measured at different time points by a spectrophotometer at 600 nm. Since PD28 cells are unable to grow on minimal medium containing maltose as the only carbon source, only cells that expressed the chimera protein in the correct orientation, where the TMD is embedded within the inner membrane and the MBP faces the periplasm, were able to utilize maltose and support cell growth. A construct with a deleted TMD (ΔTMD) served as a negative control, since the chimera protein was expected to reside in the cytoplasm and, therefore, was unable to compensate for the *malE* deficiency.

### Bacterial fractionation.

Bacterial cell fractionation was based on previously described procedures (38). Briefly, EPEC strains from an overnight culture were subcultured 1:50 in 50 ml of DMEM for 6 h at 37°C in a CO_2_ tissue culture incubator. The cultures were harvested, washed in PBS, resuspended in 1 ml of buffer A (50 mM Tris [pH 7.5], 20% [wt/vol] sucrose, protease inhibitor cocktail [Roche Applied Science], and lysozyme [100 µg/ml]), and incubated for 30 min at room temperature to generate spheroplasts. MgCl_2_ was then added to a final concentration of 20 mM, and samples were spun for 10 min at 5,000 × *g*. The supernatants containing the periplasmic fractions were collected. The pellets, which contained the cytoplasm and the membrane fractions, were resuspended in 1 ml lysis buffer (20 mM Tris-HCl, pH 7.5, 150 mM NaCl, 3 mM MgCl_2_, 1 mM CaCl_2_, and 2 mM β-mercaptoethanol with protease inhibitors). All subsequent steps were carried out at 4°C. Ten micrograms of RNase A and DNase I per milliliter was added, and the samples were sonicated (Fisher Scientific; 3 times for 15 s each). Intact bacteria were removed by centrifugation (at 2,300 × *g* for 15 min), and the cleared supernatants containing cytoplasmic and membrane proteins were transferred to new tubes. To obtain the cytoplasmic fraction, supernatants were centrifuged (in a Beckman Optima XE-90 ultracentrifuge with an SW60 Ti rotor) for 30 min at 100,000 × *g* to pellet the membranes. The supernatants containing the cytoplasmic fraction were collected, the pellets containing the membrane fractions were washed with lysis buffer, and the final pellets were resuspended in 0.1 ml lysis buffer with 0.1% SDS. The protein contents of all samples were determined using the Coomassie Plus protein assay (Thermo Scientific) before adding SDS-PAGE sample buffer with β-mercaptoethanol.

### Membrane protein extraction.

Bacterial membranes were purified as described previously (38). Details are given in Text S1 in the supplemental material.

### BN-PAGE.

Extracted membrane proteins were incubated for 5 min in the blue native (BN) sample buffer (30% glycerol with 0.05% Coomassie brilliant blue G-250) and loaded onto a 4 to 20% gradient native gel. For electrophoresis, the cathode buffer was 15 mM Bis-Tris and 50 mM Bicine (adjusted to pH 7) and the anode buffer comprised 50 mM Bis-Tris (adjusted to pH 7). The electrophoresis was carried out in ice until full separation (5 to 6 h). The gel was then subjected to Western immunoblotting with an anti-HA antibody.
