# Supplementary figures and images for: The Third Transmembrane Domain of EscR Is Critical for Function of the Enteropathogenic Escherichia coli Type III Secretion System
Source: mSphere. 2018 Jul 25;3(4):e00162-18. doi: 10.1128/mSphere.00162-18 (PMC6060343; doi:10.1128/mSphere.00162-18)

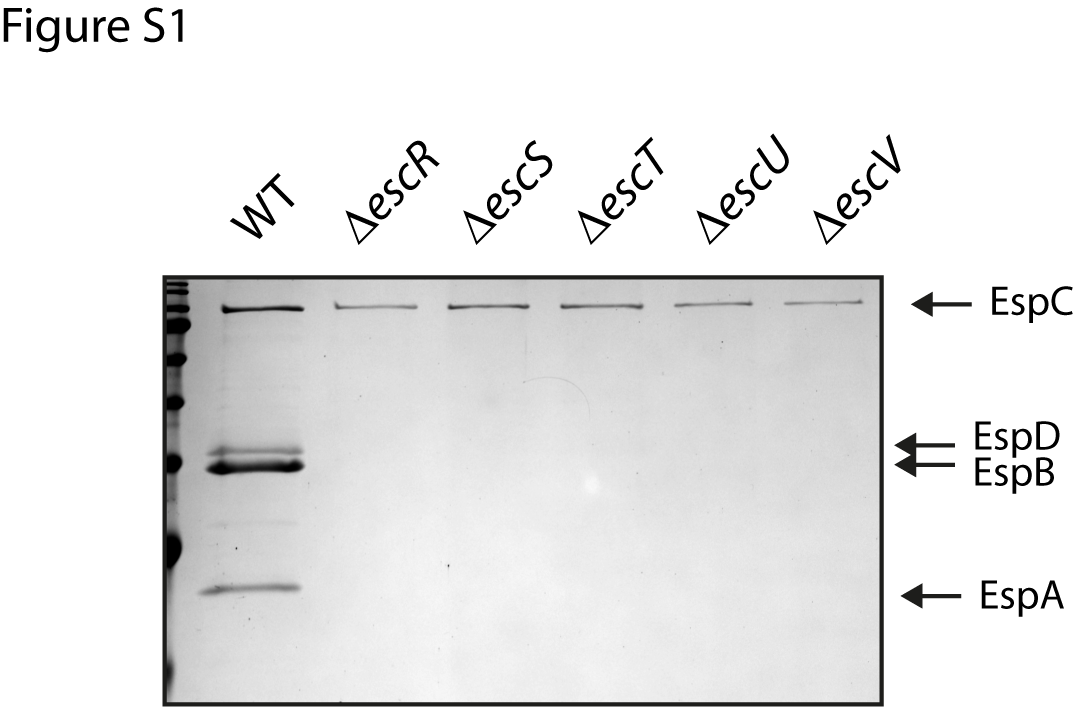

Supplement: FIG S1 [file sph004182600sf1.tif]

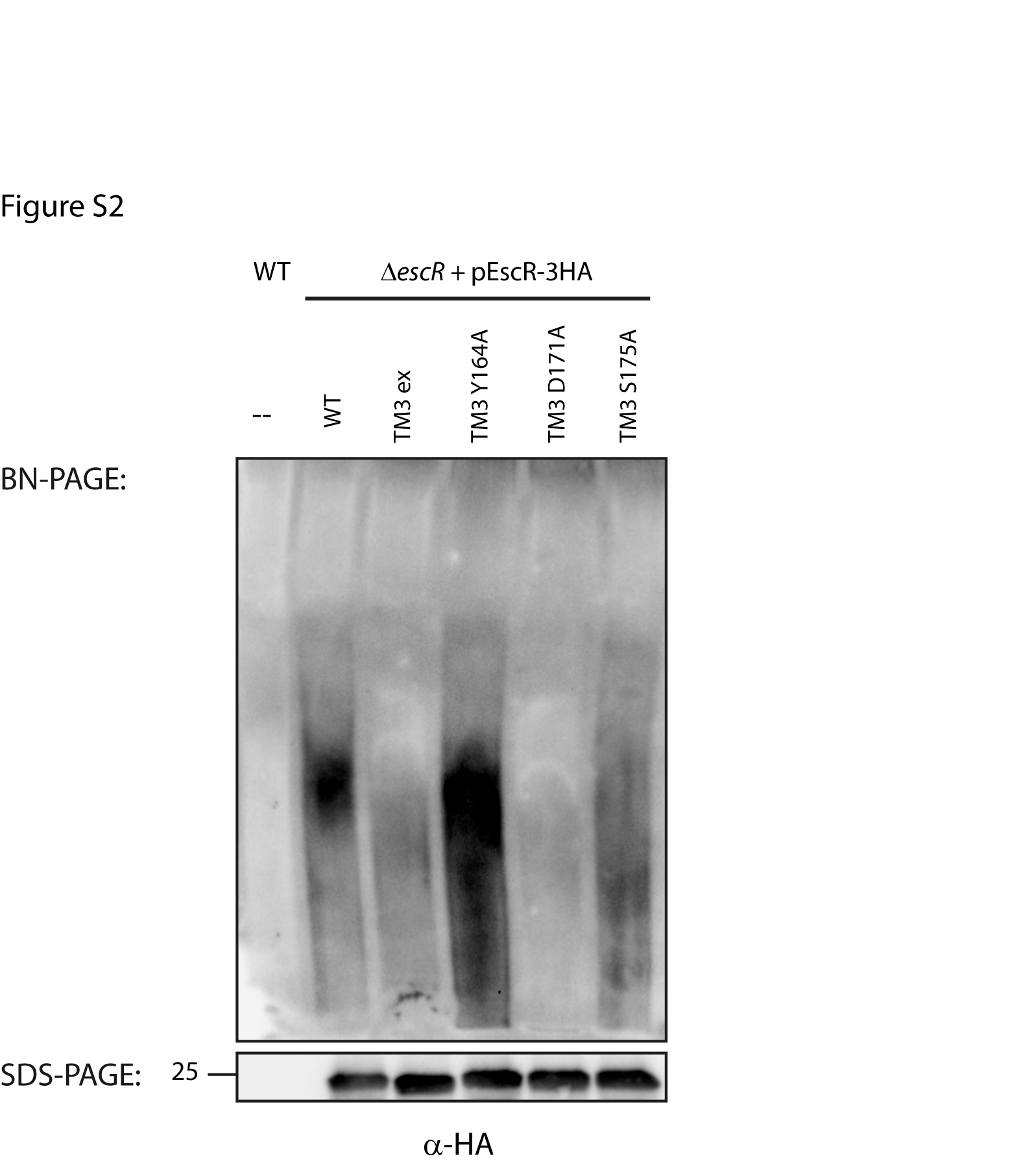

Supplement: FIG S2 [file sph004182600sf2.tif]

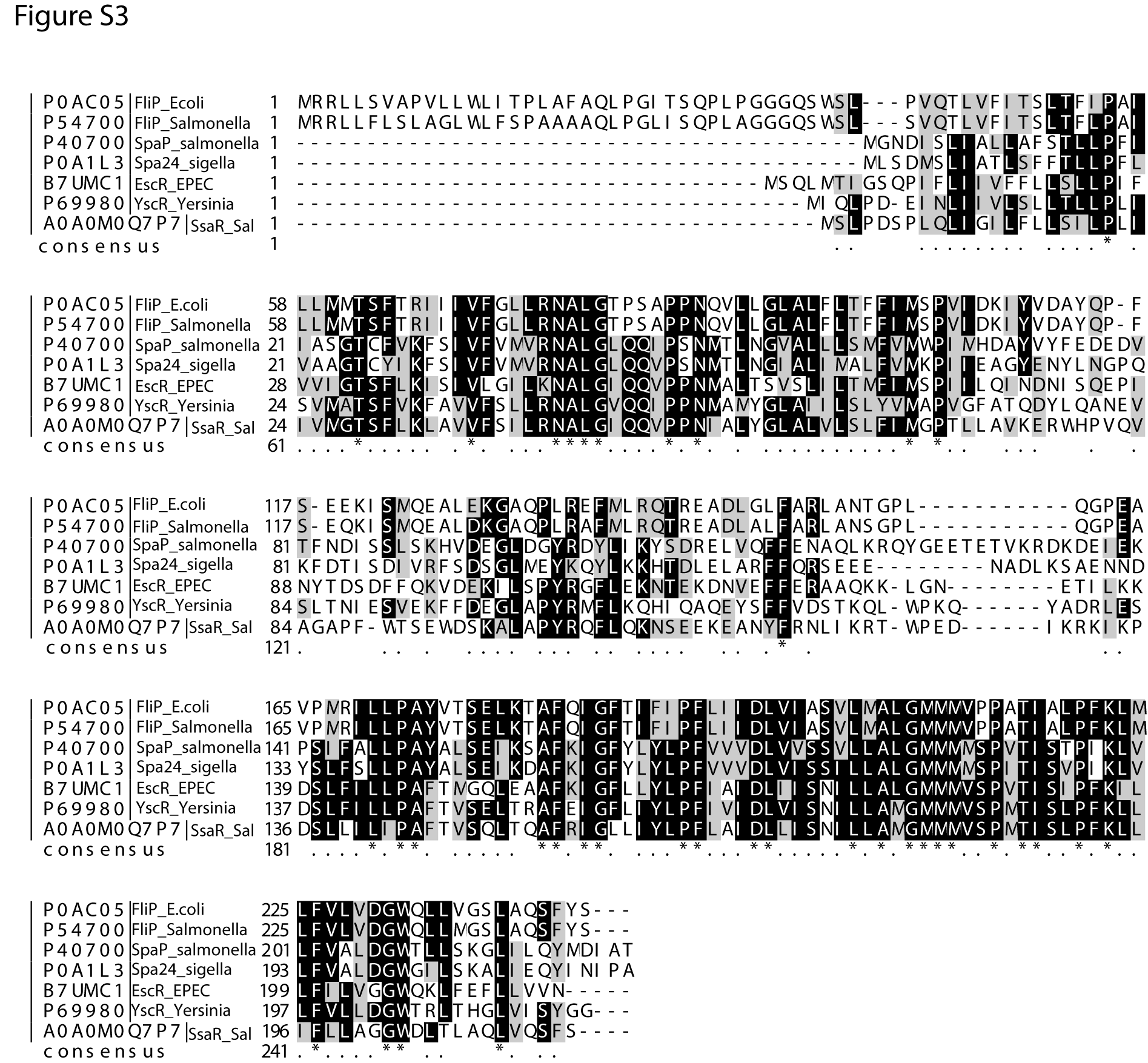

Supplement: FIG S3 [file sph004182600sf3.tif]
