# Supplementary material for: The Third Transmembrane Domain of EscR Is Critical for Function of the Enteropathogenic Escherichia coli Type III Secretion System
Source: mSphere. 2018 Jul 25;3(4):e00162-18. doi: 10.1128/mSphere.00162-18 (PMC6060343; doi:10.1128/mSphere.00162-18)
Supplement: TEXT S1 [file sph004182600s1.docx]

Supporting information

Material and methods

**Construction of single Δ*escR*, Δ*escS*, and Δ*escT*** **null mutants** - Nonpolar deletion mutants of the escR, escS and escT genes in the Sm^r^ EPEC strain E2348/69 were generated by using the sacB-based allelic exchange method (1). Briefly, two PCR fragments of the flanking regions of *escR* (0.64 and 1.62 kb, from the 5` and 3` of *escR*, respectively), *escS* (1.28 and 1.30 kb, from the 5` and 3` of *escS*, respectively), and *escT* (1.56 and 0.57 kb, from the 5` and 3` of *escT*, respectively) were generated with the corresponding primer pairs ESCR-01F/ESCR-01R, ESCR-02F/ESCR-02R, ESCS-01F/ESCS-01R, ESCS-02F/ESCS-02R, ESCT-01F/ESCT-01R, and ESCT-02F/ESCT-02R (Table 1), cloned separately into pCR2.1-TOPO (Invitrogen) and verified by DNA sequencing. The fragments corresponding to upstream sequence of the deleted genes were digested with *Sac*I/*Nhe*I while the PCR fragments of the downstream sequences of the deleted genes were digested with *Nhe*I/*Kpn*I. The fragments were then gel-purified and cloned into pRE112 digested with *Sac*I/*Kpn*I in a 3-way ligation (1). The resulting pRE112 plasmids contained the flanking regions of either escR with 86% of escR deleted, *escS* with 82% of *escS* deleted or *escT* with 88% of *escT* deleted. The plasmids were then transformed into E. coli SM10λpir to be introduced into EPEC by conjugation (2). After a sucrose selection process, EPEC colonies that were resistant to sucrose and susceptible to chloramphenicol were screened for the deletion of escR, escS or escT by PCR.

**Construction of plasmids expressing EscR-3HA and TMD3-exchanged EscR and labeling the chromosomal *escR* gene with triple HA** **–** The cloning was done by the Gibson assembly method (3, 4). Briefly, the *escR* gene was amplified from EPEC genomic DNA by using the primer pairs EscR-3HA-F/EscR-3HA-R1 and then EscR-3HA-F/EscR-3HA-R2 (Table 1), which fused a triple HA tag to the coding region of *escR*. The pSA10 was amplified with the primer pair pSA10_F/pSA10_R (Table 1). The PCR products were subjected to digestion with *Dpn*I, purified, and assembled by the Gibson assembly method. A tagged version of escR was similarly cloned into the pACYC184 plasmid (5) by amplifying the labeled gene from the pSA10 vector using the primer pair EscR-3HA-184_F/EscR-3HA-184_R (Table 1) and the pACYC184 vector using the primer pair pACYC_Gib_F/pACYC_Gib_R (Table 1). PCR products were subjected to digestion with *Dpn*I, purified, and assembled by the Gibson assembly method. The resulting constructs, pEscR_WT_-3HA in either the high copy number plasmid (pSA10) or low copy number plasmid (pACYC184), expressed a full-length EscR fused to a C-terminal triple HA tag. To label the *escR* gene with triple HA on the chromosome, we used the sacB-based allelic exchange method, mentioned above, with the pRE112 vector that carries the *escR-3HA* gene between the flanking regions of *escR*. For that purpose, we amplified *escR*-3HA from the pEscR_WT_-3HA (pSA10), using the primer pair EscR-3HA-Chr-F/ EscR-3HA-Chr-R (Table 1), and two PCR fragments of the flanking regions of *escR* (1.0 and 1.2 kb, of the chromosomal sequences 5` and 3` of *escR*, respectively) with the corresponding primer pairs ESCR-03F/ESCR-03R and ESCR-04F/ESCR-04R (Table 1). The pRE112 vector was amplified using the primer pair pRE112_Gib_F/ pRE112_Gib_R (Table 1). All four PCR products were subjected to digestion with *Dpn*I, purified, and assembled by the Gibson assembly method. The resulting pRE112 plasmid contained the flanking regions of escR with triple HA labeled escR. The plasmid was then transformed into E. coli SM10λpir to be introduced into the Δ*escR* EPEC strain by conjugation. After a sucrose selection process, EPEC colonies that were resistant to sucrose and susceptible to chloramphenicol were screened for the presence of chromosomal escR-3HA by PCR.

The TMD3-exchanged *escR* in pSA10 was generated by using the template of pEscR_wt_-3HA (pSA10). To replace the TMD3 of EscR by a TMD backbone sequence of 7-leucine-9-alanine (7L9A), the EscR 1-161 amino acid sequence was amplified by using the primer pair EscR_Gib_F/EscR_161Gib_R (Table 1) from pEscR_WT_-3HA vector. The TMD 7L9A backbone was generated by annealing the primer pair 7L9A-F/7L9A-R (Table 1) by heating the sample to 95 °C for 5 min and then decreasing the temperature to 20 °C at a rate of 5 °C/min. The EscR_1-161_ PCR fragment and the 7L9A backbone were then ligated by using overlapping sequences and amplified by using the primer pair EscR_Gib_F/EscR_7L9A_Gib_R (Table 1). Gibson assembly was conducted by amplifying the pEscR_WT_-3HA pSA10 vector with the primer pair pSA10_EsR_TM3_7L9A_F/pSA10_Gib_R (Table 1), followed by *Dpn*I treatment of the reaction and subjecting the amplified vector and the EscR_1-161_-7L9A fused PCR fragment to ligation. The resulting construct, pEscR-TM3_ex_-3HA (pSA10), expressed a TMD3 exchanged EscR with a triple HA tag at its C-terminus. Site-directed mutagenesis of Y164A, D171A and S175A within the EscR-3HA (pSA10) construct was performed using the primer pairs Y164F (EscR)/Y164R (EscR), D171F/D171R, and S175F (EscR)/S175R (EscR) (Table 1). Site-directed mutagenesis of L171D within the EscR-TM3_ex_-3HA (pSA10) construct was performed using the primer pairs L171D_F/ L171D_R. All constructs were verified by DNA sequencing.

**Construction of plasmids expressing ToxR-TMD-MBP chimera proteins** –Synthetic oligonucleotides pairs (Table 1) encoding an *Nhe*I-*Bam*HI TMD-DNA cassette of 16 core residues of the EscR TMD1, TMD2 or TMD3 (^15^IIVFFLLSLLPIFVVI^30^, ^60^TSVSLILTMFIMSPII^75^, and ^161^LLYLPFIAIDLIISNI^177^, respectively) were phosphorylated, aligned and ligated between a *toxR* transcription activator and the *malE* (encoding E. coli maltose binding protein (MBP)) within an *Nhe*I/*Bam*HI digested ToxR-MBP plasmid (6). Point mutations of Y164A, D171A and S175A within the EscR TMD3 sequence were performed using the primer pairs Y164F (ToxR)/Y164R (ToxR), D171F/D171R, and S175F (ToxR)/S175R (ToxR), respectively (Table 1). All constructs were verified by DNA sequencing.

**Membrane protein extraction** – Bacterial membranes were purified as described previously (7). Briefly, EPEC strains were subcultured 1:50 into 50 mL of pre-heated DMEM supplemented with the appropriate antibiotics and grown for 6 h in a tissue culture incubator (with 5% CO_2_) statically. The bacterial cultures were harvested by centrifugation at 3220 × g for 30 min at 4 °C. The pellet was washed with one culture volume of ice-cold PBS and pelleted by centrifugation at 3220 × g for 30 min at 4 °C. The washed pellet was resuspended in 1 mL of lysis buffer (20 mM Tris/HCl, pH 7.5, 150 mM NaCl, 3 mM MgCl_2_, 1 mM CaCl_2_, and 2 mM 2-mercaptoethanol with a protease inhibitor cocktail). Lysozyme (100 µg/mL), RNase A (10 μg/mL) and DNase I (10 μg/mL) were added to the samples and incubated on ice for 30 min. The cell suspension was then subjected to sonication (3×15 s) with a probe sonicator (Fisher Scientific). Intact cells were removed by centrifugation at 5000 × g for 15 min at 4 °C, and the cleared supernatants containing cytoplasmic and membrane proteins were transferred to new tubes. To obtain the membrane fraction, supernatants were centrifuged (in a Beckman Optima XE-90 Ultracentrifuge with a SW60 Ti rotor) for 30 min at 100000 × *g* to pellet the membranes. The supernatants, containing the soluble fractions, were discarded and the membrane pellets were washed with 2 mL lysis buffer. The membrane proteins were extracted by incubation in 1% n-Dodecyl-β-D-Maltoside (DDM) on a rotary-wheel for 60 min at 4 °C. Unsolubilized material was removed by centrifuging the samples at 20000 × g for 15 min at 4 °C. The supernatants were collected and analyzed by Blue-Native PAGE.

References

1. **Edwards RA, Keller LH, Schifferli DM.** 1998. Improved allelic exchange vectors and their use to analyze 987P fimbria gene expression. Gene **207:**149-157.

2. **Miller VL, Mekalanos JJ.** 1988. A novel suicide vector and its use in construction of insertion mutations: osmoregulation of outer membrane proteins and virulence determinants in *Vibrio cholerae* requires toxR. J Bacteriol **170:**2575-2583.

3. **Gibson DG, Benders GA, Andrews-Pfannkoch C, Denisova EA, Baden-Tillson H, Zaveri J, Stockwell TB, Brownley A, Thomas DW, Algire MA, Merryman C, Young L, Noskov VN, Glass JI, Venter JC, Hutchison CA, 3rd, Smith HO.** 2008. Complete chemical synthesis, assembly, and cloning of a *Mycoplasma genitalium* genome. Science **319:**1215-1220.

4. **Gibson DG, Young L, Chuang RY, Venter JC, Hutchison CA, 3rd, Smith HO.** 2009. Enzymatic assembly of DNA molecules up to several hundred kilobases. Nat Methods **6:**343-345.

5. **Rose RE.** 1988. The nucleotide sequence of pACYC184. Nucleic Acids Res **16:**355.

6. **Langosch D, Brosig B, Kolmar H, Fritz HJ.** 1996. Dimerisation of the glycophorin A transmembrane segment in membranes probed with the ToxR transcription activator. J Mol Biol **263:**525-530.

7. **Gauthier A, Puente JL, Finlay BB.** 2003. Secretin of the enteropathogenic *Escherichia coli* type III secretion system requires components of the type III apparatus for assembly and localization. Infect Immun **71:**3310-3319.

8. **Larkin MA, Blackshields G, Brown NP, Chenna R, McGettigan PA, McWilliam H, Valentin F, Wallace IM, Wilm A, Lopez R, Thompson JD, Gibson TJ, Higgins DG.** 2007. Clustal W and Clustal X version 2.0. Bioinformatics **23:**2947-2948.
